# Achieving Integrated Care for Older People: Shuffling the Deckchairs or Making the System Watertight For the Future?

**DOI:** 10.15171/ijhpm.2017.144

**Published:** 2018-01-03

**Authors:** Gill Harvey, Joanne Dollard, Amy Marshall, Manasi Murthy Mittinty

**Affiliations:** ^1^Adelaide Nursing School, University of Adelaide, Adelaide, SA, Australia.; ^2^Adelaide Medical School, University of Adelaide, Adelaide, SA, Australia.; ^3^Australian Research Centre for Population Oral Health, Adelaide Dental School, University of Adelaide, Adelaide, SA, Australia.

**Keywords:** Integrated Care, Older People, Multi-Morbidity

## Abstract

Integrated care has been recognised as a key initiative to resolve the issues surrounding care for older people living with multi-morbidity. Multiple strategies and policies have been implemented to increase coordination of care globally however, evidence of effectiveness remains mixed. The reasons for this are complex and multifactorial, yet many strategies deal with parts of the problem rather than taking a whole systems view with the older person clearly at the centre. This approach of fixing parts of the system may be akin to shuffling the deckchairs on the Titanic, rather than dealing with the fundamental reasons why the ship is sinking. Attempts to make the ship more watertight need to be firmly centred on the older person, pay close attention to implementation and embrace approaches that promote collaborative working between all the stakeholders involved.

Shuffling the deckchairs on the Titanic’ is a phrase commonly used to refer to futile or ineffective actions in the face of an impending crisis.^1^ Is this a fair analogy to use when discussing the issue of integrated care for older people? Or are the multiple policy initiatives to address the problem at a national and international level achieving the aim of making our health and care systems watertight so that the ship will not sink in the near future? A recent review of integrated care in England suggests not:

*“Effective integrated care has been a widespread policy ambition and commitment for many years …. While there are many examples of local leaders improving the quality and efficiency of care for people through integration, we still see too much of a gap between the national ambition and the experience of people using services in their local area”* ^2^ (p.2).

As the above quote illustrates, integrated care has been the focus of numerous policy and research endeavours over a substantial period of time, yet evidence of effectiveness remains mixed and what apparently works in one setting does not necessarily work elsewhere. Why is it such an enduring problem? Exploring this issue further, we reflect on a number of points, including: the nature of the problem and why it exists; solutions that have been tried and tested to address the problem; lessons from evidence and experiences; and how the learning could be applied to make the ship more watertight in the future.

## The Problem

Evidence suggests that many older people are ‘falling through the gaps’ and experiencing fragmented care,^3^ particularly when they live with multi-morbidity. In Australia, over 83 per cent of the 75 and over population has two or more chronic conditions,^4^ whilst in the United States of America, around half of the over 75 population is reported to have three or more chronic conditions.^5^ This population group are typically dealing with both health and functional challenges and report almost twice as many problems resulting from poorly integrated care, compared to their peers without multi-morbidity.^6^ This is because they typically see a greater number of doctors, take multiple prescription drugs, have numerous agencies involved in their care, and experience more emergency department visits and hospitalisation.^7^ In turn, this risks patient safety and contributes to poorer health outcomes, reduced quality of life and increased healthcare utilisation and costs.

Transitions of care, for example, admission to hospital or discharge from hospital to home, are particular stress points for patients and their carers^8^ and a time when effective coordination between services and care providers is paramount. However, it is also a time when communication can break down, adding to the difficulties faced by patients at the critical point of transition. This is evident in our own local example of care pathways experienced by older people who presented frequently at hospital^9^ (see Box 1). From many angles, poor coordination of care presents a significant policy and practice issue, not least because the prevalence of older people with multiple chronic conditions is continuing to increase over time.

**Box 1. The Challenge of Coordinating Care for Older People with Complex Needs – A Local Example**

We undertook an analysis of older people living within one defined metropolitan Council area in South Australia who had four or more unplanned emergency presentations at the local hospital over a 13 month period. Approximately 0.5% (n = 61) of the 60 + population met these inclusion criteria. We then conducted a more in-depth study of the care pathways of 17 of these 61 patients*, purposefully looking at different age groups, sex and those living at home or in residential aged care.

Amongst the 17 patients, there were a total of 91 hospital presentations in the 13 month period, ranging from 4 to 11 presentations per person (mean = 5.2 presentations). The time between each presentation varied from 1 to 281 days (mean = 44.3 days). Eighty-two percent of presentations were made by ambulance and 75% of presentations resulted in hospital admission. Sixteen of the 17 older people presented for the same or similar condition more than once.

In relation to communication between care providers, 21% of separation summaries were delayed by more than 7 days (range 11 to 73 days) and only 19% of patients discharged home had a discharge plan written for them by health care staff.

*The sub-sample of 17 patients was purposively identified to achieve representation of men and women, different age categories, living situation (alone or with partner/family) and place of residence (home or residential aged care).

## Policies and Initiatives Directed at Solving the Problem

The example in Box 1, whilst on a small scale, presents a fairly typical scenario of the issues faced – vulnerable older people bouncing between hospital and the community, often within short periods of time and with gaps in communication between the various agencies and people involved in their care. Patients themselves lacked written information about their admission and necessary information to manage at home. Numerous initiatives have been tried and tested to address the problems associated with fragmented care, both at a policy level and through empirical study. Comparing integrated care initiatives across seven countries, Wodchis and colleagues highlight competing policy drivers, such as the primary focus of improving user experience and independence versus a focus of reducing hospital utilisation and associated costs.^5^ The latter is particularly evident in the Unites States where a legislative approach was adopted with the Affordable Care Act, which introduced the Hospital Readmission Reduction Program. This program imposes financial penalties on hospitals that have higher than expected 30 day readmission rates for particular conditions.^10^

Just as the motives for improving integrated care may vary, so do the approaches adopted to improve integration. These include attempts at integration that adopt a vertical, horizontal, professional or organizational perspective at either a micro, meso or macro level of the health system.^5^ Such attempts at improving integration are often centred on a strong primary care foundation, for example, the Patient Centred Medical Home (PCMH) concept in the United States^11^ and similar initiatives in other countries, such as the introduction of Health Care Homes in Australia,^12^ and Better, Sooner, More Convenient health care in the community (New Zealand).^13^ Yet in their international evaluation, Wodchis and colleagues noted that general practitioners were rarely part of the core team, because they proved difficult to engage in data sharing due to a combination of factors, such as intensive workloads, their independent status and related funding arrangements.^5^

Some evaluations of integrated care indicate improved care outcomes and reduced hospital utilisation and cost^11,14^; however, significant challenges of implementation are reported in relation to managing transformational change, and working within pluralistic delivery systems, with different cultures, multiple stakeholders and different mechanisms of funding and governance. This reflects the reality of most attempts to improve the integration of care across boundaries such as acute, primary and community care, which require engagement and buy-in of a range of different stakeholder groups, including healthcare professionals, carer organisations, local government, patients and families. Successful approaches are typically initiated by bottom-up innovation, driven by local needs and supported by relevant policies and resources, but with common elements such as a single point of entry, holistic care assessment, comprehensive care planning and a care coordinator role.^5^

## Learning From Evidence and Experiences to Date

Comparing the aspirations for integrated care to the reality of what has been achieved, there are clear examples of success, often reported as exemplar case studies of how to make it happen. However, leveraging from isolated examples of success to wider, sustained improvement at a health system level continues to pose a significant challenge. As the international review reports, even the most successful examples have had to *‘work against the grain’* (p.10) of how health systems and organizations typically function,^5^ often with some additional financial or legal measures in place to support them. Even when such special measures are in place, there may be disagreement as to their utility and validity, as for example, in the case of the US Hospital Readmission Reduction Program, with some commentators challenging the 30 day readmission metric, given that evidence suggests perhaps only around a quarter of readmissions are actually preventable because they are driven by patient and community-level factors.^10^

What is most obvious from evidence and experiences to date is that there is no one-size-fits-all approach to integrated care, hence the need to pay close attention to processes of implementation. This requires the development of tailored and locally relevant integrated care programs that take account of barriers and enablers within the contextual setting. It is also important to recognise that the meaning of integrated care can vary according to whose definition is centre-stage. At a policy and service delivery level, there is an economic imperative to reduce the costs associated with poorly integrated care. However, this may not adequately address what matters most to patients and their families, as highlighted by the work of organisations such as National Voices in the United Kingdom. Their research with patients, service users and carers highlighted a strong desire for coordination (not necessarily organizational integration) and care, with less concern about the source of the care.^15^ Patients emphasized *relational* aspects of care as the most important for a positive experience. This may be in contrast to the way in which policy-makers and service providers think and act, as demonstrated by recent research on the differences between lay and medical discourse in care coordination.^16^ This research undertaken with older people with multiple chronic co-morbidities shows how patient narratives emphasize the impact of their condition on their everyday life, including feelings of vulnerability and disempowerment. However, this was not typically captured in the medical record, nor was important contextual information, such as the patient’s living situation, the presence and role of informal carers, functional impairments and experience of pain. As the authors note, this indicates a lack of agreement between the patient and the care provider about the priority and practical significance of health issues and a failure to capture important information about the level of patient ability to coordinate their own care.

## Applying the Learning Moving Forwards

Back to our metaphor of the Titanic, are there some key messages we need to take on board to make our systems more watertight and improve integrated care for older people? We would suggest there are several important points to consider. Firstly, we need to pay attention to implementation. Technical solutions alone will not be sufficient to solve the problem of fragmented and uncoordinated care. Rather, we need to acknowledge the complexity of the issues we are dealing with and pull on available evidence to develop locally relevant, bottom-up solutions. This means engaging with older people, their families and carers, health and social care providers and building the networks and relationships that will lay the foundations for effective coordination of care. In turn, this requires a system-wide view that addresses entire pathways of care, rather than simply ‘shuffling the deckchairs’ and looking for solutions within particular sectors, such as acute hospitals, primary care or community services. Secondly, we need to keep centred on the person, listening to what older people want and need and adopting a holistic view that embraces health, functional and social issues. Thirdly, we need to consider co-production approaches, such as experience-based design and participatory action research. With their focus on a whole systems perspective that embraces dynamic interaction and local adaptation, these engaged approaches to research could support the development and implementation of context-relevant, person-centred integrated care.^17,18^

Pursuing more engaged, adaptive and person-centred solutions to the problem of fragmented care will require leadership, vision and political will, against the backdrop of increasing demand and constrained resources for health and social care. Whether the ship is actually big enough to cope with the demands placed on it remains to be seen.

## Acknowledgements

We gratefully acknowledge funding from The Hospital Research Foundation (THRF), Woodville, SA, Australia that is supporting our research into improving the integration of care for older people.

## Ethical issues

Not applicable.

## Competing interests

Authors declare that they have no competing interests.

## Authors’ contributions

All authors have been involved in research that contributes to our views in this editorial. GH drafted the original version of the manuscript. All authors contributed revisions and approved the final version.

## Authors’ affiliations

^1^Adelaide Nursing School, University of Adelaide, Adelaide, SA, Australia. ^2^Adelaide Medical School, University of Adelaide, Adelaide, SA, Australia. ^3^Australian Research Centre for Population Oral Health, Adelaide Dental School, University of Adelaide, Adelaide, SA, Australia.
